# Draft genomes of *Cronobacter sakazakii* strains isolated from dried spices bring unique insights into the diversity of plant-associated strains

**DOI:** 10.1186/s40793-018-0339-6

**Published:** 2018-11-29

**Authors:** Hyein Jang, Jungha Woo, Youyoung Lee, Flavia Negrete, Samantha Finkelstein, Hannah R. Chase, Nicole Addy, Laura Ewing, Junia Jean Gilles Beaubrun, Isha Patel, Jayanthi Gangiredla, Athmanya Eshwar, Ziad W. Jaradat, Kunho Seo, Srikumar Shabarinath, Séamus Fanning, Roger Stephan, Angelika Lehner, Ben D. Tall, Gopal R. Gopinath

**Affiliations:** 1 Center of Food Safety and Applied Nutrition, U. S. Food and Drug Administration, 8301 Muirkirk Road, Laurel, MD 20708 USA; 2 Department of Nutrition and Food Technology, Jordan University of Science and Technology, Irbid, 22110 Jordan; 3 Center for One Health, College of Veterinary Medicine, Konkuk University, Seoul, 05029 South Korea; 4 UCD Centre for Food Safety, School of Public Health, Physiotherapy & Population Science, University College, Dublin, Ireland; 5 WHO Collaborating Centre for Cronobacter, Belfield, Dublin 4, Ireland; 6 Institute for Food Safety and Hygiene, University of Zurich, Zurich, Switzerland

**Keywords:** *Cronobacter sakazakii*, WGS, Draft Genomes, Plant-origin, Dried Spices

## Abstract

*Cronobacter sakazakii* is a Gram-negative opportunistic pathogen that causes life- threatening infantile infections, such as meningitis, septicemia, and necrotizing enterocolitis, as well as pneumonia, septicemia, and urinary tract and wound infections in adults. Here, we report 26 draft genome sequences of *C. sakazakii*, which were obtained from dried spices from the USA, the Middle East, China, and the Republic of Korea. The average genome size of the *C. sakazakii* genomes was 4393 kb, with an average of 4055 protein coding genes, and an average genome G + C content of 56.9%. The genomes contained genes related to carbohydrate transport and metabolism, amino acid transport and metabolism, and cell wall/membrane biogenesis. In addition, we identified genes encoding proteins involved in osmotic responses such as DnaJ, Aquaproin Z, ProQ, and TreF, as well as virulence-related and heat shock-related proteins.

Interestingly, a metabolic island comprised of a variably-sized xylose utilization operon was found within the spice-associated *C. sakazakii* genomes, which supports the hypothesis that plants may serve as transmission vectors or alternative hosts for *Cronobacter* species. The presence of the genes identified in this study can support the remarkable phenotypic traits of *C. sakazakii* such as the organism’s capabilities of adaptation and survival in response to adverse growth environmental conditions (e.g. osmotic and desiccative stresses). Accordingly, the genome analyses provided insights into many aspects of physiology and evolutionary history of this important foodborne pathogen.

**Electronic supplementary material:**

The online version of this article (10.1186/s40793-018-0339-6) contains supplementary material, which is available to authorized users.

## Introduction

*Cronobacter* species, formerly known as *Enterobacter sakazakii*, are a group of opportunistic foodborne bacterial pathogens [1, 2]. The genus *Cronobacter* is comprised of seven species: *C. sakazakii*, *C. malonaticus*, *C. turicensis*, *C. muytjensii*, *C. dublinensis*, *C. universalis*, and *C. condimenti* [2, 3]. These re-emerged pathogens cause severe meningitis, septicemia, or necrotizing enterocolitis in neonates and infants and pneumonia, septicemia, and urinary tract and wound infections in adults [4–7]. Of the seven species, the primary pathogen is *C. sakazakii*; the status of *Cronobacter*, as a pathogen, was elevated to an international public health concern when contaminated samples of powdered infant formula (PIF) or follow-up formula (FUF) were recognized by the food safety community, after linking its presence to several neonatal meningitis outbreaks [8, 9, 10]. It is well-defined now that contamination of reconstituted, temperature-abused PIF occurs both intrinsically and extrinsically; the main reservoir(s) and routes(s) of contamination have yet to be established, however [11]. Furthermore, reports from numerous surveillance studies have shown that *Cronobacter* species are found in a variety of foods including dried foods (spices, herbs, flour, and cereals) and fresh ready-to-eat vegetables [12–15]. This increasing body of evidence suggests that plants may serve as a reservoir [16, 17]. Moreover, linking the epidemiology of adult cases to consumption of PIF is difficult to explain [5–7], suggesting that there are still unknown sources, such as other foods which may be involved in causing adult infections. Although occurrences of *Cronobacter* species in plant- origin foods are increasingly being reported, relatively less genomic information is available [18, 19]. Here, we describe the draft genome sequences of 26 *C. sakazakii* strains isolated from dried spices which were obtained from the USA, the Middle East, China, and the Republic of Korea.

## Organism information

### Classification and feature

**Fig. 1:**
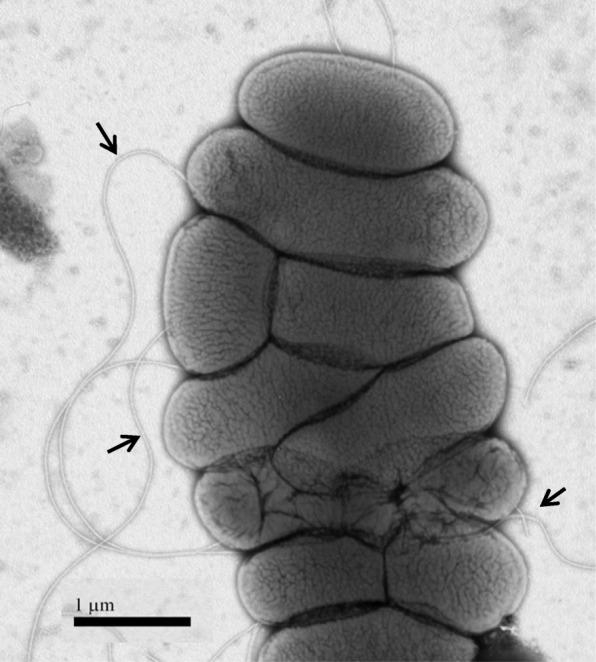
Transmission electron photomicrograph of a typical *Cronobacter sakazakii* strain (ES632) grown on Trypticase soy agar supplemented with 1% sodium chloride, and incubated at 37 °C for 22 h. The cells were negatively stained with 0.5% sodium phosphotungstate (pH 6.8). Note the presence of numerous peritrichously expressed flagella (arrow). Bar represents 1 μm

The strains described in this report were obtained through various surveillance studies reported by Gopinath et al. [18], Jaradat et al. [20], and Chon et al. [21]. *C. sakazakii* is a Gram-negative, non-sporulating, and mesophilic, facultatively anaerobic bacterium (Kingdom Domain: Bacteria) that belongs to the phylum *Proteobacteria,* class *Gammaproteobacteria,* order *Enterobacterales**,* within the family *Enterobacteriaceae*. *C. sakazakii* cells are rod-shaped measuring approximately 3 by 1 μm when the cells are in the exponential growth phase; the cells are motile by peritrichously-expressed flagella (Fig. 1). The species type strain is ATCC 29544^T^ (strain synonyms: CDC 4562–70; DSM 4485; NCTC 11467, and WDCM 00214), which was isolated from a child’s throat with whooping cough in 1970 by the Tennessee State Health Department, Nashville, TN, USA. Originally described as a yellow pigmented *E. cloacae* by Urmenyi and Franklin [22], the bacterium was later reclassified by Farmer et al. as *Enterobacter* *sakazakii* in 1980 [23], and then redefined as *Cronobacter* by Iversen et al. [2] after aligning the different biogroups described by Farmer et al. [23] into separate species epithets. Iversen et al. [2] characterized the new genus into six species groups based on a polyphasic approach utilizing both DNA-DNA hybridization and phenotypic analyses. Joseph et al. [3], then described *C.* *condimenti* and realigned the previously recognized *Cronobacter* genomospecies 1 with the new species epithet, *C. universalis*.

Phenotypically, it is very challenging to assign species identities to *Cronobacter* species based on classic biochemical reactions routinely used to characterize members of the family *Enterobacteriaceae*; Iversen et al. [2] have summarized these concerns. They assigned biogroups 1–4, 7, 8, 11, and 13 to the *C. sakazakii* epithet [2]. Typically, *C. sakazakii* strains will give a positive result in tests for the utilization of putrescine, turanose, maltitol, lactulose, 1–0-methyl a- D-glucopyranoside, palatinose, cisaconitate and 4-aminobutyrate. The utilization of myo-inositol is variable among strains and a small number of strains (less than 5%) can utilize malonate [2].

*Cronobacter* species also represent a group of bacteria that are highly resistant to desiccation [24–28, 29, 30].

*Cronobacter* species are ubiquitous in nature, and molecular typing schemes have been very helpful in both epidemiological and surveillance investigations. One of the most useful schemes is based on a DNA-sequence-typing (ST) method using a seven-locus MLST scheme which is maintained at http://www.pubmlst.org/cronobacter [31, 32, 33]. Recently Gopinath et al. [18] demonstrated that *C. sakazakii* strains possessing the ST64 allelic profile also contain a nine gene, 7.7 kb malonate utilization operon which shares sequence homology with operons possessed by *C. turicensis* and *C. universalis*. These results support the original findings of Iversen et al. [2] that projected that ~ 5% of *C. sakazakii* strains can utilize malonate, a trait well recognized to be present in the other six *Cronobacter* species. There have been over 230 *C**.* *sakazakii* STs identified and 11% of ~ 1606 *C. sakazakii* strains stored within the *Cronobacter* PubMLST site are from clinical samples [31]. *C. sakazakii* ST64 strains are phylogenetically related to strain *C. sakazakii* strain GP1999, a ST145 strain which was isolated from a tomato plant’s rhizoplane/rhizosphere continuum [16, 17], as well as, to other strains obtained during surveillance studies of dried plant foods, PIF and dairy powder production facility environments, spice, milk powder, and mushroom samples located throughout the USA, Europe, the Middle East, the Republic of Korea, and China [18–21]. The general features of the strains reported in the present study are shown in Table 1 which includes five ST64 strains: AS (Allspice) 2, AS4, AS13, AS15, and Jor172 which were obtained from spice samples from the USA, the Republic of Korea, China, and Jordan. Strains representing 12 other STs are also incorporated into this report, including strains representing STs like the meningitis ST4 clone and other clinically relevant STs: ST1, ST8, ST3, ST13, ST21, ST31, ST40, ST99, ST219, ST226, and a recent new ST: ST643 [19].

**Table 1:** Classification and general features of *C. sakazakii* strains used in this study

MGS ID	Property	Term	Evidence Code^a^
	Classification	Domain: Bacteria	
		Phylum: Proteobacteria	
		Class: Gammaproteobacteria	
		Order: Enterobacteriales	
		Family: *Enterobacteriaceae*	
		Genus: *Cronobacter*	
		Species: *sakazakii*	
		Strains: MOD1_AS-2, MOD1_AS-4, MOD1_AS-13, MOD1_AS-15, MOD1_Jor20, MOD1_Jor22, MOD1_Jor44, MOD1_Jor93, MOD1_Jor96, MOD1_Jor103, MOD1_Jor146, MOD1_Jor148, MOD1_Jor151, MOD1_Jor154, MOD1_Jor172, MOD1_Jor173, MOD1_Jor178, MOD1_Jor183, MOD1_KW3, MOD1_KW13, MOD1_O21–13, MOD1_O21–16, MOD1_O26–1, MOD1_O26–4, MOD1_O23mB, MOD1_788569	
	Gram stain	Negative	TAS [2]
	Cell shape	Rod-shaped	TAS [2]
	Motility	Motile by peritrichous flagella	TAS [2]
	Sporulation	Non-sporulating	TAS [2]
	Temperature range	6 to 45 °C	TAS [2]
	Optimum temperature	37 °C	TAS [2]
	pH range	pH 5 to 10	TAS [2]
	Carbon source	α-D-glucose, β-D-fructose, D-galactose, trehalose, D-mannose, α-melibiose, sucrose, raffinose, maltotriose, maltose, α-lactose, 1–0-methyl α/β-galactopyranoside, cellobiose, β-gentiobiose, 1–0-methyl β-D-glucopyranoside, aesculin, L-arabinose, D-xylose, glycerol, D-mannitol, L-malate, D-glucuronate, D-galacturonate, 2-keto-D-gluconate, N-acetyl D-glucosamine, arbutin, DL-α-glycerol-phosphate, dihydroxyacetone, D-ribose, L-lyxose, pyruvic acid, D-gluconate, DL-lactate, succinate, fumarate, DL-glycerate, D-glucosamine, L-aspartate, L-glutamate, L-proline, D-alanine, L-alanine and L-serine.	TAS [2]
MIG5–6	Habitat	Environment, Eukaryotic plant-origin, Human	TAS [2]
	Energy source	Chemoheterotrophic	TAS [2]
MIG6–3	Salinity	Grows up to 10% NaCl	TAS [2]
MIG5–22	Oxygen requirement	Facultatively anaerobic	TAS [2]
MIG5–15	Biotic relationship	Eukaryotic plant-origin, Human	TAS [2]
MIG5–14	Pathogenicity	Human pathogen	TAS [2]
MIG5–23	Isolation	Bacteriological Analytical Manual, ISO/TS 22964:2017	TAS [62–64]
MIG5–4	Geographic location	USA, Europe, Asia, Central America, South America	TAS [2]
MIG5–5	Sample collection	Plant-origin	TAS [2]
MIG5–4.1	Latitude	variable	TAS [2]
MIG5–4.2	Longitude	variable	TAS [2]
MIG5–4.4	Altitude	variable	TAS [2]

^a^Evidence codes: *TAS* Traceable author statement (i.e., a direct report exists in the literature). These codes are from the Gene Ontology project [42]

## Genome sequencing information

### Genome project history

This extended genome report describes draft genomes of twenty-six *C. sakazakii* strains which were obtained from various spice samples. This work is part of a larger study focused on exploring the microbial diversity of *C. sakazakii* strains which are associated with foods of plant- origin such as spices; Table 2 describes the project information and its association with minimum information about a genome sequence (MIGS) utilizing its version 2.0 compliance criteria [34].

**Table 2:** Minimum information about a genome sequence (MIGS); project information for the 26 spice- associated *C. sakazakii* strains

MIGS ID	Property	Term
MIGS 31	Finishing quality	Improved high-quality draft
MIGS-28	Libraries used	Illumina Nextera XT, pair-end
MIGS 29	Sequencing platforms	Illumina MiSeq
MIGS 31.2	Fold coverage	50X
MIGS 30	Assemblers	de novo assembly, CLC Genomics Workbench version 9.0
MIGS 32	Gene calling method	RAST annotation server [33]; JGI, NCBI
	Locus Tag	See Table 3
	Genbank ID	See Table 3
	GenBank Date of Release	2018/03/07
	GOLD ID	SEE Table 3
	BIOPROJECT	PRJNA258403 (*Cronobacter* GenomeTrakr Project, FDA-CFSAN)
	Project relevance	Food Safety, source attribution

### Growth conditions and genomic DNA preparation

Frozen bacterial cultures were stored at − 80 °C in Trypticase soy broth (BBL, Cockeysville, MD) supplemented with 1% NaCl (TSBS) and 50% glycerol, and were streaked onto agar plates containing *Enterobacter sakazakii* Chromogenic Plating Medium (ESPM, R&F Products; Downers Grove, IL) followed by incubation overnight at 37 °C. Typical *Cronobacter*- like colonies (blue-black to blue-gray colored, raised colonies) were chosen to inoculate TSBS broth cultures (5 ml) which were incubated at 37 °C, shaking at 150 rpm for 18 h. Bacterial DNA was extracted and purified using a Qiagen Qiacube instrument and its automated technology (QIAGEN Sciences; Germantown, MD) as described previously and according to the manufacturer’s instructions [16, 18, 19, 35, 36].

### Genome sequencing and assembly

For WGS analysis of the strains, the concentration of each strain’s DNA was then determined using a Qubit Fluorometric spectrophotometer (Life Technologies, Thermo Fisher Scientific; Wilmington, DE). DNA samples were diluted with sterile nuclease-free deionized water (molecular biology grade, Thermo Fisher Scientific, Waltham, MA) to a final concentration of 0.2 ng/μl. Whole-genome sequencing was performed using a MiSeq benchtop sequencer (Illumina, San Diego, CA, USA), utilizing either 500 or 600 cycles of paired-end reads (Illumina). FASTQ datasets were de novo assembled with CLC Genomics Workbench version 9.0 (CLC bio, Aarhus, Denmark). The paired end libraries were generated and sequenced in conjunction with the Nextera XT DNA sample preparation guide on the Illumina Miseq instrument (Illumina; San Diego, CA) [16, 18, 19].

### Genome annotation

Sequence data for each strain was uploaded onto the Rapid Annotation Subsystems Technology (RAST) server for annotation [37]. The genomes were also submitted to the Department of Energy Joint Genome Institute (Walnut Creek, CA) through the annotation submission portal of the NCBI prokaryotic genome annotation pipeline (PGAP) with its best- placed reference protein set GeneMarkS+ application. Table 3 shows each strain’s source, geographic locale, genome size, topology, %G + C content, number of CDS, sequence type (ST), NCBI accession number, GOLD analysis project identification number, and locus tag which are captured for each spice-associated strain under the umbrella NCBI GenBank BioProject PRJNA258403 which is a FDA-CFSAN *Cronobacter* GenomeTrakr project [38, 39]. EggNOG analysis was also used to verify functional gene annotations and to help identify clusters of orthologous groups (COGs) categories [40].

**Table 3:** Draft genomes, source, geographic locale, genome size, topology, %G + C content, No. of CDS, sequence type (ST), accession numbers, GOLD project ID, and locus tag of strains captured under the FDA-CFSAN *Cronobacter* GenomeTrakr NCBI BioProject PRJNA258403 and used in this study

Strain Name	Source	Geographic Locale	Genome Size (kb)	Topology	G + C content (%)	No. of CDS	ST	NCBI Accession no.	GOLD Analysis Project ID^b^	Locus tag
MOD1_Jor173	Unknown Spice	Jordan	4403	Circular	56.9	4030	1, CC1	PVCG00000000	Ga0259519	PVCG01
MOD1_Jor146	Liquorice	Jordan	4409	Circular	56.9	4059	3, CC3	PVMV00000000	Ga0259523	PVMV01
MOD1_Jor96	Fennel	Jordan	4667	Circular	56.6	4337	4, CC4	PVCE00000000	Ga0259516	PVCE01
MOD1_Jor148	Unknown Spice	Jordan	4573	Circular	56.8	4251	4, CC4	PVCF00000000	Ga0259517	PVCF01
MOD1_Jor154	Unknown Spice	Jordan	4392	Circular	56.9	4064	4, CC4	NITP00000000	Ga0260550	NITP01
MOD1_Jor178	Chamomile	Jordan	4787	Circular	56.4	4409	4, CC4	PVBV00000000	Ga0259520	PVBV01
MOD1_KW13	Dried Garlic	Republic of Korea	4493	Circular	56.9	4176	13, CC13	NITD00000000	Ga0260553	NITD01
MOD1_Jor183	Unknown Spice	Jordan	4326	Circular	56.9	3934	21, CC21	NITN00000000	Ga0260551	NITN01
MOD1_788569	Siberian Ginseng, *Eleutherom sentiocosus* Root Powder	China	4503	Circular	56.8	4162	31, CC31	PVCL00000000	Ga0259506	PVCL01
MOD1_KW3	Dried Hot Pepper	Republic of Korea	4372	Circular	56.9	4042	40, CC40	NITH00000000	Ga0260552	NITH01
MOD1_AS-2	Allspice	USA	4306	Circular	57.0	3987	64, CC64	PVCH00000000	Ga0259508	PVCH01
MOD1_AS-4	Allspice	USA	4297	Circular	57.0	3975	64, CC64	PVCI00000000	Ga0259509	PVCI01
MOD1_AS-13	Allspice	USA	4312	Circular	57.0	3980	64, CC64	PVCJ00000000	Ga0259510	PVCJ01
MOD1_AS-15	Allspice	USA	4313	Circular	57.0	3983	64, CC64	PVCK00000000	Ga0259511	PVCK01
MOD1_Jor172	Unknown Spice	Jordan	4331	Circular	57.0	4012	64, CC64	NCWD00000000	Ga0260555	NCWD01
MOD1_O21_16	Oregano	USA	4407	Circular	57.0	4071	99, CC99	PVSQ00000000	Ga0260560	PVSQ01
MOD1_O26_1	Oregano	USA	4408	Circular	57.0	4071	99, CC99	PVBX00000000	Ga0259522	PVBX01
MOD1_O21_13	Oregano	USA	4375	Circular	57.0	4059	219, CC155	PVBW00000000	Ga0259521	PVBW01
MOD1_O23mB	Oregano	USA	4339	Circular	56.9	3991	226, CC8	PVBZ00000000	Ga0259507	PVBZ01
MOD1_O26_4	Oregano	USA	4338	Circular	56.9	3972	226, CC8	PVBY00000000	Ga0260554	PVBY01
MOD1_Jor20	Unknown Spice	Jordan	4468	Circular	56.7	4117	226, CC8	PVCA00000000	Ga0259512	PVCA01
MOD1_Jor22	Chamomile	Jordan	4469	Circular	56.7	4112	226, CC8	PVCB00000000	Ga0259513	PVCB01
MOD1_Jor44	Unknown Spice	Jordan	4482	Circular	56.9	4133	8, CC8^a^	PVCC00000000	Ga0259514	PVCC01
MOD1_Jor151	Unknown Spice	Jordan	4489	Circular	56.9	4142	8, CC8^a^	PVMW00000000	Ga0259518	PVMW01
MOD1_Jor93	Unknown Spice	Jordan	4331	Circular	57.1	3973	643	PVCD00000000	Ga0259515	PVCD01
MOD1_Jor103	Unknown Spice	Jordan	4425	Circular	57.0	4014	643	NITR00000000	Ga0260549	NITR01

^a^Six exact matches (100% homology) of the allelic profiles (allele profile number in parentheses) for the *Cronobacter* MLST genes: (8) *fusA*, (7) *glnS*, (5) *gltB*, (8) *gyrB*, (15) *infB* and (10) *pps,* and the closest match of these strains in the MLST database is strain 2274, MLST ID 1390 (alias, L1). The closest ST match is ST8, CC8 except that the allelic profile number for *atpD* was 121 for these strains which differs from the reported allelic profile number 11 for this ST.

^b^JGI IMG/MER study ID number is Gs0133658

## Genome properties

A summary of the genome statistics for the 26 plant-origin *C. sakazakii* strains is provided in Table 4 and information on each individual strain is given in Additional file 1: Table S1. De novo assembly of the genomes resulted in an average total genome length of 4393 kb with a range of 4052 to 4716 kb observed among the genomes. The average total number of coding regions (CDS) was determined to be 3898 kb with a CDS range of 3779 to 4160 kb observed among the genomes (take note: that the JGI IMG annotation pipeline identified 3151 genes which were assigned to COGs). The average G + C content of strains was 56.9% with a range of 56.4 to 57.1% observed among the genomes. These values are similar to those reported for other strains of plant-origins curated at NCBI [16, 18, 19, 35, 36]. Using the JGI IMG annotation pipeline, it was possible to identify an average of 4207 predicted genes (range: 4090-4541) among the 26 genomes of which 4055 (3937 to 4383) genes putatively encoded for proteins (which constituted ~ 96% of all genes). One-hundred pseudogenes (range: 73–157 genes), and 151 RNA genes (range: 142–162 genes) were also identified; 3877 genes possessed identifiable Pfam domains, while ~ 413 genes encoded proteins possessing predicted signal peptides. Lastly, approximately 994 genes encoded for predicted proteins with a function that could be assigned to a transmembrane protein.

**Table 4:** Summary of the genome statistics of the 26 *C. sakazakii* strains evaluated in this study^a^

Attribute	Value	Range	% of Total
Genome size (kb)	4393	4052-4716	100.0
DNA coding (kb)	3898	3779-4160	88.3
Number of DNA G + C bases (kb)	2510	2438-2664	56.9
DNA scaffolds	46.2	23–100	100.0
Total genes	4207	4090-4541	100.0
Protein coding genes	4055	3937-4383	96.4
RNA genes	151.6	142–162	3.6
Pseudo genes^d^	100.6	73–157	- ^c^
Genes in internal clusters	887.1	829–962	21.0
Genes assigned to COGs	3,151^b^	3101-3251	74.9
Genes with Pfam domain	3877	3595-3879	87.4
Genes with signal peptides	413.5	403–436	9.8
Genes with transmembrane proteins	994.6	978–1038	23.7
CRISPR repeats^d^	2.6	2–4	-^c^

^a^Data was obtained from the JGI IMG pipeline. Note: Genome statistics for each individual strain is shown in Additional file 1: Table S1

^b^The number of genes assigned to COGs by NCBI was 3902 compared to the value (3151 genes) assigned by the JGI IMG pipeline

^c^NCBI pipeline did not have the % total for the CRISPR repeats and pseudo genes

^d^Data was obtained from the NCBI, https://www.ncbi.nlm.nih.gov/nuccore

The distribution of each strain’s proteins into COG functional categories [41, 42] is summarized in Table 5 and information for individual strains is shown in Additional file 2: Table S2 and Additional file 3: Table S3. Two of the 23 COG categories, namely those assigned to Codes B and R which are designated for proteins associated with chromatin structure and dynamics, and general function prediction were not assigned. Notably, 4% of the proteins were not found in any COGs. Unfortunately, the COG category identified in this study which possessed the highest number of assigned proteins was COG category S which is allocated for proteins (~ 23%) designated as functionally uncharacterized. Protein COG categories which were associated with the top 11 other COG categories (within parentheses) were: (G) carbohydrate transport and metabolism (8.3%); (K) transcription (7.8%); (E) amino acid transport and metabolism (7.2%); (M) cell wall/membrane biogenesis (6.3%); (P) inorganic ion transport and metabolism (6.0%), (C) energy production and conversion (5.3%), (J) translation, ribosomal structure and biogenesis (4.5%); (L) replication, recombination and repair (4.3%); (O) post-translational modification, protein turnover, and catabolism (3.8%); and (H) coenzyme transport and metabolism and (T) signal transduction mechanisms (both 3.8%). That fact that these *C. sakazakii* strains’ genomes possessed genes encoding a large proportion of putative proteins (~ 35% of the remaining ~ 77% of their COG assigned proteins) which were dedicated to carbohydrate, amino acid, cell wall/membrane biogenesis, inorganic ion transport and metabolism, post-translational modification/protein turnover, catabolism, and coenzyme transport/metabolism supports the consensus hypothesis that these organisms have evolved to represent one of the most desiccant- resistant bacterial species found to date [24–28, 29, 30].

**Table 5:** Summary of the average number of genes and percentage of each genome representing each COG functional category associated with the 26 *C. sakazakii* strains evaluated in this study^a^

Code	Value	%age	Description
J	177	4.5	Translation, ribosomal structure and biogenesis
A	1	0.0	RNA processing and modification
K	304	7.8	Transcription
L	168	4.3	Replication, recombination and repair
B	0	0.0	Chromatin structure and dynamics
D	46	1.2	Cell cycle control, Cell division, chromosome partitioning
V	54	1.4	Defense mechanisms
T	149	3.8	Signal transduction mechanisms
M	245	6.3	Cell wall/membrane biogenesis
N	75	1.9	Cell motility
U	64	1.6	Intracellular trafficking and secretion
O	146	3.8	Posttranslational modification, protein turnover, chaperones
C	206	5.3	Energy production and conversion
G	323	8.3	Carbohydrate transport and metabolism
E	281	7.2	Amino acid transport and metabolism
F	102	2.6	Nucleotide transport and metabolism
H	148	3.8	Coenzyme transport and metabolism
I	84	2.1	Lipid transport and metabolism
P	235	6.0	Inorganic ion transport and metabolism
Q	46	1.2	Secondary metabolites biosynthesis, transport and catabolism
R	0	0.0	General function prediction only
S	894	22.9	Function unknown
–	154	4.0	Not in COGs

The total is based on the total average number of protein coding genes (3902) for the genome. ^a^Note: A summary of the total number of COG alleles per strain is shown in Additional file 2: Table S2. Individual strain’s genome statistics is shown in Additional file 3: Table S3

## Insights from the genome sequence

### Plasmids

Comparative RAST analysis of the draft assemblies with that of the virulence plasmid, pESA3 (131,196 bp in size [37]), shown in Additional file 4: Table S4, revealed the presence of coding sequences for the predicted alleles of the pESA3-like, RepFIB virulence plasmid originally described by Franco et al. [43]. pESA3-like plasmids contain a common backbone set of alleles represented by the plasmid origin of replication gene, *repA*, an ABC iron transporter gene cluster (identified by the presence of *eitA*) and a Cronobactin (an aerobactin-like siderophore) gene cluster (identified by the presence of *iucC*). Prototypical *C. sakazakii* strain BAA-894 also possesses plasmidborne gene sequences for a *Cronobacter* plasminogen activator gene (*cpa*), genes encoding an ~ 17-kbp type six secretion system (T6SS) and, in approx. 20% of *C. sakazakii* strains (however, not found in BAA-894), possess genes of the ~ 27-kbp gene filamentous hemagglutinin (FHA) gene cluster represented by the presence of *fhaB* [44, 43]. Interestingly, results of PCR analysis of the strains reported in the present study, shown in Table 6, revealed that all of the strains were PCR-positive for *repA*, *cpa*, *eitA*, and *iucC*. All of the strains were also PCR-positive for the T6SS’s IntLeft (IntL) gene locus, but only seven, 11, and three of the strains were PCR-positive for the other three T6SS alleles (*vgrG*, R end, IntR). These results suggest that the T6SS gene cluster is highly variable in these strains, similar to what Franco et al. [43] and Yan et al. [45, 46] had previously reported. In addition, six of the strains were PCR-positive for *fhaB*, signifying that these strains possess the FHA gene cluster. Only one of the strains was PCR-positive for pESA2-like plasmids, while five of the strains were PCR-positive for the *C. turicensis*-like pCTU3 plasmid which was identified by Stephan et al. [47]. RAST analysis was used to determine if any of the 26 plant-origin strains harbored the small cryptic CSK29544_2p-like plasmid which has been found in other *C. sakazakii* strains such as *C. sakazakii* strain SP291 (CSK29544_2p is homologous to pSP291–3), a highly persistent environmental strain found associated with an Irish PIF manufacturing facility [45, 46]. According to the *C. sakazakii* NCBI website (https://www.ncbi.nlm.nih.gov/genome/genomes/1170?), the species type strain, *C. sakazakii* 29544^T^ harbors three plasmids CSK29544_1p (pESA3-like virulence plasmid, 93,905 bp in size), CSK29544_2p (a small cryptic plasmid, 4938 bp in size), and CSK29544_3p (a pESA2- like conjugative plasmid, 53,457 bp in size). CSK29544_2p contains five genes encoding for a methyl-accepting chemotaxis protein, a hypothetical protein and a plasmid mobilization relaxosome protein cluster, MobCABD. Our analysis showed that none of the strains harbored this plasmid (data not shown).

**Table 6:** Prevalence and distribution of pESA3 alleles associated with the virulence plasmid and pESA2/pCTU3 plasmids harbored by 26 spice-associated *C. sakazakii* isolates

No. of *C. sakazakii*	pESA3/pCTU1 (incFIB, *repA*)	No. of isolates with the indicated plasmidotype^a^									
		*cpa*	T6SS				FHA	Iron acquisition		Other plasmids^b^	
		*cpa*	Int L	*vgrG*	R end	Int R	*fhaB*	*eitA*	*iucC*	pESA2/pCTU2	pCTU3 (incH1)
26	26 (100)	26 (100)	26 (100)	7 (27)	11 (42)	3 (12)	6 (23)	26 (100)	26 (100)	1 (4)	5 (21)

^a^Numbers within parentheses are the percentage of PCR-positive strains for each gene locus in relation to the total number of plasmid- harboring spice-associated *C. sakazkaii* strains

^b^Only 24 strains were analyzed by PCR for presence of pESA2 and pCTU3 (MOD1_788569 and MOD1_O123mB strains were not analyzed). Therefore, the percent positive for pESA2 and pCTU3 were calculated using a total number of 24 strains

### Chromosomal traits

**Fig. 2:**
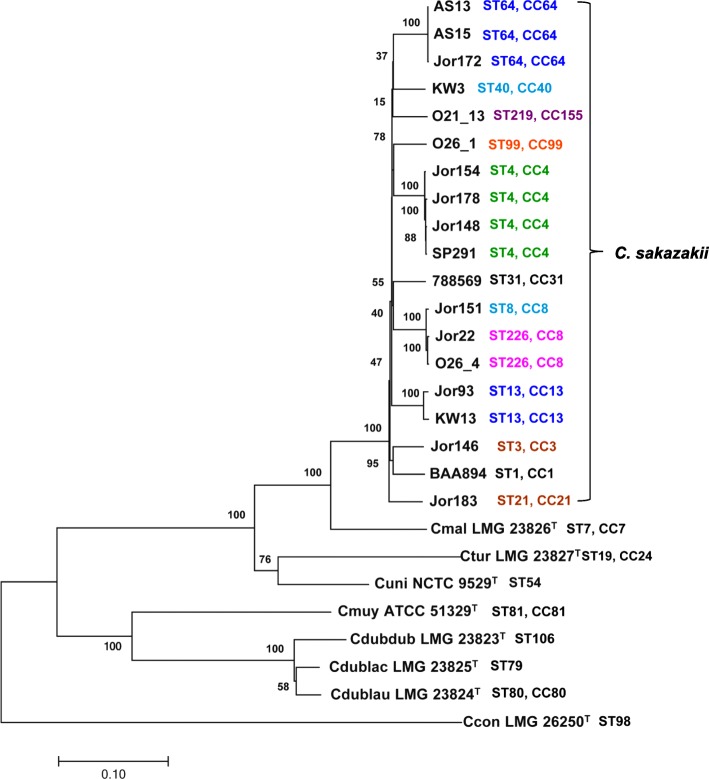
Phylogenetic analysis of *Cronobacter sakazakii* strains isolated from spices, compared with eight representative *Cronobacter* species strains (marked with superscripted ‘T’ after each strain’s name). NCBI GenBank Accession numbers of type strains: *C. malonaticus* LMG 23826^T^ (NZ_CP013940), *C. turicensis* LMG 23827^T^ (NC_013282), *C. universalis* NCTC 9529^T^ (NZ_CP012257), *C. muytjensii* ATCC 51329^T^ (NZ_CP012268), *C. dublinensis* subsp. *dublinensis* LMG 23823^T^ (NZ_CP012266), *C. dublinensis* subsp. *lactaridi* LMG 23825^T^ (NZ_AJKX00000000), *C. dublinensis* subsp. *lausannensis* LMG 23824^T^ (NZ_AJKY00000000), and *C. condimenti* LMG 26250^T^ (NZ_CP012264). Whole genome SNP analysis was carried out using kSNP3 software [48]. The phylogenetic tree was built using neighbor-joining method [65] and the evolutionary distances were computed using the Maximum Composite Likelihood method [66] available on MEGA7 phylogenetic suite [67]. The bootstrap values obtained from 500 bootstrap replicates are reported as percentages at the nodes [68]. Sequence type (ST) information was obtained by uploading each strain’s genome assembly to the *Cronobacter* MLST website (http://pubmlst.org/cronobacter/) after which the ST information was manually overlaid onto the tree with different color. Note that the phylogeny among the strains followed ST evolutionary lineages. The scale bar indicates 0.10 substitutions per nucleotide position

Next generation genome sequencing of the different *Cronobacter* species revealed a species-level bidirectional divergence which is hypothesized to be driven by niche adaptation [35]. Figure 2 illustrates this phylogenetic divergence, using the kSNP3 tool [48], of the strains reported in this study with representative strains of each species. The phylogeny among these strains followed similar sequence type evolutionary lineages which were reported by Chase et al. [36] and Gopinath et al. [18]. Furthermore, *Cronobacter* possess a diversity of remarkable features which support the organism’s capability to survive under severe environmental growth conditions such as xerotolerant econiches confined to the production of dried foods, such as PIF [35, 29, 30]. The physiological mechanisms of desiccation survival are thought to involve both primary and secondary desiccation responses; and involve the efflux of various sugars such as trehalose and other osmoprotectants [29, 30]. Genomically, several genes involved in osmotic responses were found within these spice-associated strains; furthermore, these genes were shown by Srikumar et al. [30] to be transcriptionally highly up-regulated in *C. sakazakii* cells grown under xerotolerant growth conditions. For example, DnaJ and DnaK, (Additional file 3: Table S3) in strain MOD1_O23mB, represented by locus tags: C5975_08705 and C5975_08710 are two co- expressed chaperone proteins which are classified in COG O and were found in all of the strains analyzed in this study. DnaJ participates actively in the response to hyperosmotic and heat shock by preventing the aggregation of stress-denatured proteins and acts in association with DnaK and GrpE (locus tag C5975_09365). DnaJ is considered to be the nucleotide exchange factor for DnaK and may function as a thermosensor. Unfolded proteins bind initially to DnaJ. It is also hypothesized that DnaJ, DnaK, and GrpE act together in the replication of plasmids through activation of initiation proteins. Another protein, Aquaporin Z (classified in COG M, represented here as an example in strain MOD1_O23mB (locus tag: C5975_14540) Additional file 3: Table S3), was found in all strains and is a porin-like channel protein that permits osmotically driven movement of water in both directions. It is thought to be involved in osmoregulation and in the maintenance of cell turgor pressure during volume expansion in rapidly growing cells. It is thought that Aquaporin Z opens in response to the stretch forces in the membrane lipid bilayer and that it may also participate in the regulation of osmotic pressure changes within the cell during osmotic stress. Thus, Aquaporin Z mediates rapid entry or exit of water in response to abrupt changes in osmolarity. Aquaporin Z is also a member of the major intrinsic protein (MIP) superfamily which functions primarily as water-selective membrane channels that transport water, small neutral molecules, and ions out of and between cells. Still another protein, ProQ (as example, locus C5975_18900 in strain MOD1_O23mB in Additional file 3: Table S3), is classified in COG T; and is a protein that is a structural element that influences the osmotic activation of the proline/betaine transporter ProP at a post-translational level. It also acts as a proton symporter that senses osmotic shifts and responds by importing osmolytes such as proline, glycine betaine, stachydrine, pipecolic acid, ectoine and taurine into the cell. ProP is thought to have a dual role in that it serves the cell as both an osmosensor and an osmoregulator which is available to participate in the bacterial osmoregulatory response [29, 30]. The channel opens in response to the stretch forces in the membrane lipid bilayer and may also participate in the regulation of osmotic pressure changes within the cell. Other proteins such a TreF (an alpha, alpha-trehalase, MOD1_O23mB locus C5975_10755, COG G, Additional file 3: Table S3) was found and is thought to provide cells with the ability to utilize trehalose under high osmolarity growth conditions by splitting it into glucose molecules that can subsequently be taken up by the phosphotransferase-mediated uptake system. Another set of proteins encoded by the *mdoHGC* operon (COG P, MOD1_O23mB locus C5975_17925, C5975_17930, C5975_17940 in Additional file 3: Table S3), which is involved in the biosynthesis of osmoregulated periplasmic glucans (OPGs), was found to be highly up-regulated in *C. sakazakii* grown under xerotolerant growth conditions [30]. The roles of the OPGs are complex and vary considerably among bacteria, but OPGs are thought to be a part of a signal transduction pathway(s) and are thought to indirectly regulate genes involved in virulence. The total number of OPGs increases when the osmolarity growth conditions decreases [49]. In general, EggNOG analysis identified 10 proteins per strain that were involved in the osmotolerance response. Another group of chaperone-like proteins which these *C. sakazakii* strains possessed are also annotated as heat shock proteins, and consist of IbpA (C5975_06750), DiaA (C5975_07735), and HtpX (C5975_18890), and Hsp15 (C5975_00700, COG M). There were in general between 11 and 17 heat shock-related proteins found by EggNOG analysis. Other sets of proteins found associated with these strains include 22–27 fimbriae proteins, however no curli proteins were found. There were 23–28 different efflux pump-associated proteins including proteins involved with the efflux or transport of threonine, homoserine lactone (locus tag C5975_00275), p-hydroxybenzoic acid (locus tag C5975_07280), glutathione-regulated potassium (locus tag C5975_00475, C5975_00480, C5975_08855, C5975_08860, KefGFCB), RND efflux (C5975_02520, Transporter), proteins associated with heavy metal efflux of nickel/cobalt (C5975_13445, RcnB), cobalt/magnesium (C5975_08880, ApaG), and manganese ions (C5975_18840, MntP), sugar efflux (C5975_13720, SetB), and multidrug resistance (MdtA, MdtH, MdtD). There were on average 5–13, 1–10, 15–20 proteins that were annotated as integrases, transposases, and recombinase-like proteins, respectively. All of these genes have been observed in other *C. sakazakii* genomes [16, 18, 19]. Interestingly, there was a large difference (11–63) in the number of phage-associated proteins among the strains. For example *C. sakazakii* strain Jor96 possessed phage proteins annotated for lambda, GP49-like, P2, Mu, and cp-933 k phages. Lastly there was also a wide difference in the number of both toxin-antitoxin type I and type II toxin-antitoxin family proteins found among the genomes; examples include type I toxin-antitoxin system hok family toxin and type II toxin- antitoxin systems such as RelE/ParE, RelE/DinJ, and HipA families.

Among the spice-associated *C. sakazakii* strains, 4 to 7 hemolysin- related proteins were identified. For example *C. sakazakii* strain MOD1_Jor93 possessed six alleles encoding for hemolysin-related proteins, such as four COG category U (intracellular trafficking and secretion) genes. A hemolysin secretion/activation protein homologous to the ShlB/FhaC/HecB family of alleles was found in MOD1_Jor93 (C5940_08565, Additional file 3: Table S3). This Pfam annotated allele shares homology with a group of sequences that are related to ShlB from *Serratia* *marcescens* [50]. It is hypothesized that ShlB is an outer membrane protein possibly involved in either a Type V or a two-partner secretion system where it functions to secrete and activate a ShlA type hemolysin. The activation of ShlA is thought to occur during secretion when ShlB imposes a conformational change in the inactive hemolysin to form the active protein. Though ShlA was not found in MOD1_Jor93, this protein was found in MOD1_Jor20 (C5932_21600).

There were three proteins defined as COG category S (function unknown) which included a hemolysin expression modulating protein, a putative hemolysin, and COG1272, a predicted membrane hemolysin III which Cruz et al. previously described [51].

Other virulence-related proteins included MsgA (analogous with a DNA damage- inducible protein, DinI family protein). Every genome possessed genes for this protein. The same protein is found in *Salmonella enterica subsp. enterica*. It is thought that MsgA in *Salmonella* is required for intramacrophage survival and seems to be independent of the PhoP regulon [52]. Other virulence factor-like proteins found were ImpE and SrfB [46].

Xylose and arabinose account for more than 30% of the total sugars in agricultural residues and in fact, Xylose is the second most abundant sugar in nature besides glucose and primarily exists as D-xylose [53]. However, it is usually found as a polymeric component of plant cell wall matrix polysaccharides such as xylans, e.g., arabinoxylans, hemicellulose (xylan, glucuronoxylan), and xyloglucan [53]. Complex interactions are thought to exist between human pathogens and a plant’s indigenous microflora, including phytopathogens, which are associated with fresh produce [53]. *Xanthomonas* pathogens such as *X. campestris* pathovars cause diseases of agronomic importance throughout the world; examples include black rot disease in crucifers such as cauliflower, cabbage, garden cress, bok choy, broccoli, and brussel sprouts; and in fact these pathovars can affect all cultivated brassicas. Also, *X. campestris* pv. *vesicatoria* (now reclassified as *X. euvesicatoria*), causes bacterial spot disease on pepper and tomato plants, and *X. campestris* pv. *malvacearum* (now *X. axonopodis* pv. *malvacearum*), causes angular leaf spot of cotton [54, 55]. These phytopathogens possess a number of plant cell wall-degrading enzymes (as part of the carbohydrate utilization with TonB-dependent outer membrane transporter system regulon, CUT), which are secreted by a type II secretion system (T2SS) and are required for virulence and pathogenesis. These pathogens also possess two major xylanase-related genes, *xynA* and *xynB*, which could influence biofilm formation and virulence by weakening the plant cell wall structure through degradation causing the release of nutrients during plant colonization [54]. A xylanolytic-like system, ubiquitous in lignocellulose-degrading bacteria, is also found in *E. coli* [56], and thought to play important roles in biofilm formation, nutrient uptake and adaptation of these *Proteobacteria* to the plant phyllosphere [56]. Functional metagenomic findings reported by Carter et al. [57] and transcriptional analyses suggest that *E. coli* O157:H7 competes with spinach indigenous microflora for essential macronutrients which is thought to lead to its ability to contaminate spinach [57, 58].

**Fig. 3:**
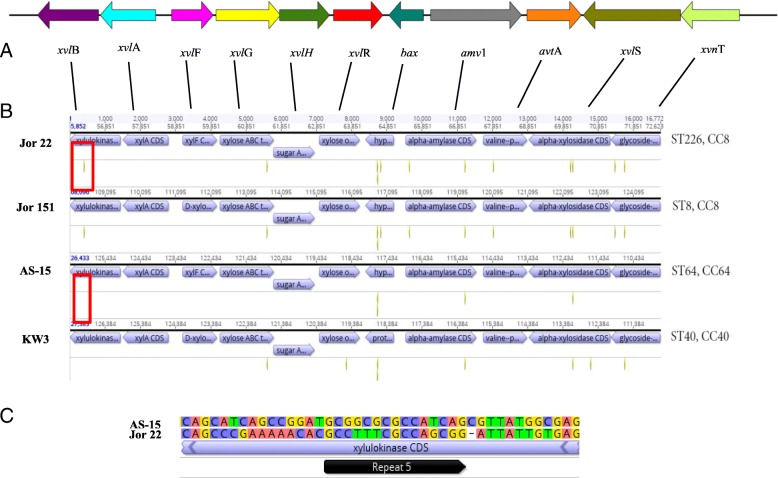
Schematic map made using XPlas, Map DNA for Mac OS XAp (http://www.iayork.com) showing the annotated xylose utilization operon from MOD1_AS-15 *C. sakazakii* strain (**a**). Xylose utilization operon for *C. sakazakii* strains MOD1_Jor22, _Jor151, AS-15,_ KW3 which were extracted from PairWise Alignments using Geneious (https://www.geneious.com/) showing the identical sequence repeat regions which are associated with each gene of the operon and captured using the identical repeat sequence region function in Geneious (**b**). Repeat regions are denoted by bronzed colored lines below each operon gene. These repeated regions are also shown in Additional file 5: Table S5. **c** Nucleotide sequence alignment captured in Geneious for repeat region five in *xylB* for MOD1_AS-15 and MOD-1_Jor22 showing the presence of the repeat region in MOD1_Jor22 (56,266 to 56,280, see red box). Note that *bax* can contain two to three identical repeat regions which suggest that this is an important highly regulated gene. *bax* has been shown to induce cell apoptosis of *Arabidopsis* protoplast cells through reactive oxygen independent and dependent processes namely DNA fragmentation, increased vacuolation, and loss of plasma membrane integrity [61]

A xylose utilization operon (average size of ~ 16,771 bp; 11 genes) which possessed a G + C content of 54.9%, was found among the spice-associated *C. sakazakii* strains. A map of the operon for *C. sakazakii* strain MOD1_AS15 is shown in Fig. 3a. The operon consists of the following genes: *xylA* (xylose isomerase, locus tag C5965_02230), *xylB* (xylulose kinase, locus tag C5965_02235), *xylF* (D-xylose ABC transporter substrate binding protein*,* locus tag C5965_02225), *xylG* (xylose ABC transporter ATP binding protein*,* locus tag C5965_02220), *xylH* (a sugar ABC transporter permease*,* locus tag C5965_02215), which is part of the ABC transporter complex XylFGH. This latter complex is involved in D-xylose uptake, *xylR* (an AraC-like xylose operon transcription regulator*,* locus tag C5965_02210), *bax* (an ATP- ribonucleoside binding protein, locus tag C5965_02205), an α-amylase gene (*amy1*, locus tag C5965_02200), a valine-pyruvate transaminase gene (*avtA*, locus tag C5965_02195), *xylS* (an α- xylosidase gene*,* locus tag C5965_02190), and a proposed α-*xynT* (glycoside-pentoside- heuronide family transporter, locus tag C5965_02185). Outside of the xylose utilization operon are other xyloside uptake genes and genes encoding degradation enzymes, such as a second *xynT* (a proposed *β-xynT,* locus tag C5965_04340), *xynB* (a β-xylosidase*,* locus tag C5965_04335), and *xylE* (a proton-sugar symporter (locus tag C5965_09300). This shares significant homology with *xylE* of *E.coil*, which is a member of the major facilitator superfamily (MFS) of transporters) possessed by *E. coli* and other bacteria [56]. The genomic structure of the *Cronobacter* xylose utilization operon was similar to that found in *E. coli* strain K-12 (strain MG1655; GenBank assembly accession: GCA_000005845; RefSeq assembly accession:GCF_000005845) except that two genes present in the *Cronobacter* xylose operon, *xylS* and α- *xynT* are missing from within the operon in *E. coli* strain MG1655 which resulted in ~ 13,041 bp sized operon. Additionally, there was a size difference (ranging from 16,340 to 16,790 bp) observed among the operons possessed by the twenty-six *C. sakazakii* strains, and there were four strains which differed in that *bax* and the α-xynT were either truncated or duplicated.

Previously we reported the presence of a xylose utilization operon in *C. sakazakii* strain GP1999, which was isolated from a tomato’s rhizoplane/rhizosphere continuum [16]. Furthermore the xylose utilization operon was found in 29 other *C. sakazakii* strains [19] which were obtained from foods of plant origin and dried-food manufacturing environments, supporting the hypothesis that plants may be the ancestral econiche for *Cronobacter* spp., as posited by Schmid et al. [17] and Joseph et al. [32]. Among these strains, we also observed differences in size of the operon [19]. In comparison, the CUT-like xylose utilization operon possessed by *X. axonopodis* pv. *citri* strain AW12879 (NCBI GenBank assembly accession number: GCA_000349225; RefSeq assembly accession: GCF_000349225) comprises a total of 13 genes and was 25,382 bp in size. Noteworthy, within this operon, an IS3 family transposase was located next to an α- glucosidase gene. Additional differences found were the presence of a TonB-dependent receptor gene and a LacI family transcriptional regulator gene (data not shown).

In the current report, we show the G + C content of a 17, 970 bp region upstream and a 17,422 bp region downstream of the *C. sakazakii* xylose utilization operon possessed G + C contents of 58.1 and 59.6%, respectively (data not shown). This change in G + C content suggests that the *Cronobacter* xylose utilization operon may be a predicted genomic (GI) or metabolic island [59]. Because bacterial genomes evolve through re-combinational events such as mutations, rearrangements, or horizontal gene transfer, we looked for clusters of genes of known or predicted GIs. Genomic islands were historically classified into distinct subtypes depending on the functions they encoded: e.g., symbiotic islands, metabolic islands, fitness islands, pathogenicity islands, or antibiotic resistance islands [60]. However, such G + C content change was not seen in the genomes of the *E. coli* and the *X. axonopodis* pv. *citri* strains. As shown in Fig. 3b, and similar to the xylose operon of *E. coli* strain MG1655 a number of sequence repeats (two in the case of MG1655) were located throughout the *Cronobacter* xylose operon (up to six sequence repeat regions were observed in some strains) suggesting that these are binding sites for regulatory proteins or that they may be evidence of past transpositions. For any one strain, there were multiple sequence repeats found. Table 7 shows examples of the various inverted repeats, palindromes and direct repeats observed in two *C. sakazakii* strains MOD1_Jor151 and MOD1_Jor173. Inverted and direct repeats were sometimes found in two different genes within the same strain (MOD1_Jor151 *amy1* and *xylS* or *xylG* and *xynT*); while palindromic sequence was found in *bax* of MOD1_Jor151. Occasionally, the size of the sequence repeat varied between 15 or 16 bases (which are the default parameters for the sequence repeats finder algorithm within Geneious). Finally, the location of the sequence repeats and type of sequence repeats found among the strains generally followed sequence type evolutionary lines with the exception of ST4 strains (MOD1_Jor148, MOD1_Jor154) and ST643 strain (MOD1_Jor103) which possessed different palindromic sequences which were associated with hypothetical protein or bax. Additional file 5: Table S5 shows the location of each the identical repeat regions within each strain’s xylose utilization operon. It should be noted that other palindromic inverted repeats (IR) of 10 to 13 nucleotides, separated by a 10-bp spacer, forming a stem-loop structure, are found on the virulence plasmids, pESA3 and pCTU1. Furthermore, Franco et al. [43] showed that a conserved pCTU1 region was located upstream of this IR, while the *Cronobacter* plasminogen activator locus on pESA3 was located downstream from this sequence repeat. Also, the upstream flanking gene seen in the *Cronobacter* xylose utilization operon was identified as a hydrolase and the downstream flanking gene was identified as DUF- 2778. These two genes and their locations were conserved throughout the 26 spice-associated *C**.* *sakazakii* genomes. Figure 3c shows an alignment of a *xylB* gene that has the IR repeat region from strain MOD1_Jor22 compared to strain MOD1_AS15 which lacks this repeat region. Note that *bax* can contain two to three identical repeat regions suggesting that this is a highly regulated gene. Bax has been shown to induce cell apoptosis of *Arabidopsis* protoplast cells through reactive oxygen independent and dependent processes namely DNA fragmentation, increased vacuolation, and loss of plasma membrane integrity [61]. Together, these results suggest that there is a virulence factor function to Bax and that the *Cronobacter* xylose utilization operon may be a predicted metabolic island.

**Table 7:** Summary of inverted repeat, palindrome, and direct repeat present in *C. sakazakii* strains MOD1_Jor151 and MOD1_Jor173 genomes^a^

Type of repeats	Strain	Gene	Sequence
IR^b^	MOD1_Jor151 (108,510-108,524)c	*xylB*	GCCTTTCGCCAGCGG…
	MOD1_Jor151 (117,747-117,761)	*amy1*	…CCGCTGGCGAAAGGC
	MOD1_Jor151 (120,139-120,154)	*avtA*	GACAAATGGCAGCCAG…
	MOD1_Jor151 (122,314-122,329)	*xylS*	…CTGGCTGCCATTTGTC
	MOD1_Jor151 (119,330-119,345)	*amy1*	GCTGTTTCGCGAAGGC…
	MOD1_Jor151 (122,381-122,396)	*xylS*	…GCCTTCGCGAAACAGC
P	MOD1_Jor151 (116,843-116,858)	*bax*	CATGGTCG CGACCATG…
	MOD1_Jor151 (116,843-116,858)	*bax*	…CATGGTCG CGACCATG
DR	MOD1_Jor151 (113,704-113,719)	*xylG*	TCACCAGCTGGTGCAG…
	MOD1_Jor151 (123,862-123,877)	*xynT*	TCACCAGCTGGTGCAG…
	MOD1_Jor151 (116,936-116,950)	*bax*	GTAACGCTTCGCGAT…
	MOD1_Jor151 (123,587-123,601)	*xynT*	GTAACGCTTCGCGAT…
	MOD_Jor173 (74,114-74,128)	*xylR*	TGTGCTGGTGCCGCC…
	MOD_Jor173 (81,051-81,065)	*xylS*	TGTGCTGGTGCCGCC…

^a^Genome assemblies were analyzed using the sequence repeat finder algorithm within Geneious. These two examples represent the various sequence repeat permutations found among the 26 spice-associated strains. For specific locations of the sequence repeats for each stain please refer to Additional file 5: Table S5

^b^Abbreviations: *IR* Inverted repeat, *P* Palindrome, *DR* Direct repeat

^c^Numbers within the parenthesis refer to the start and end base position of sequence repeats within Geneous

**Fig. 4:**
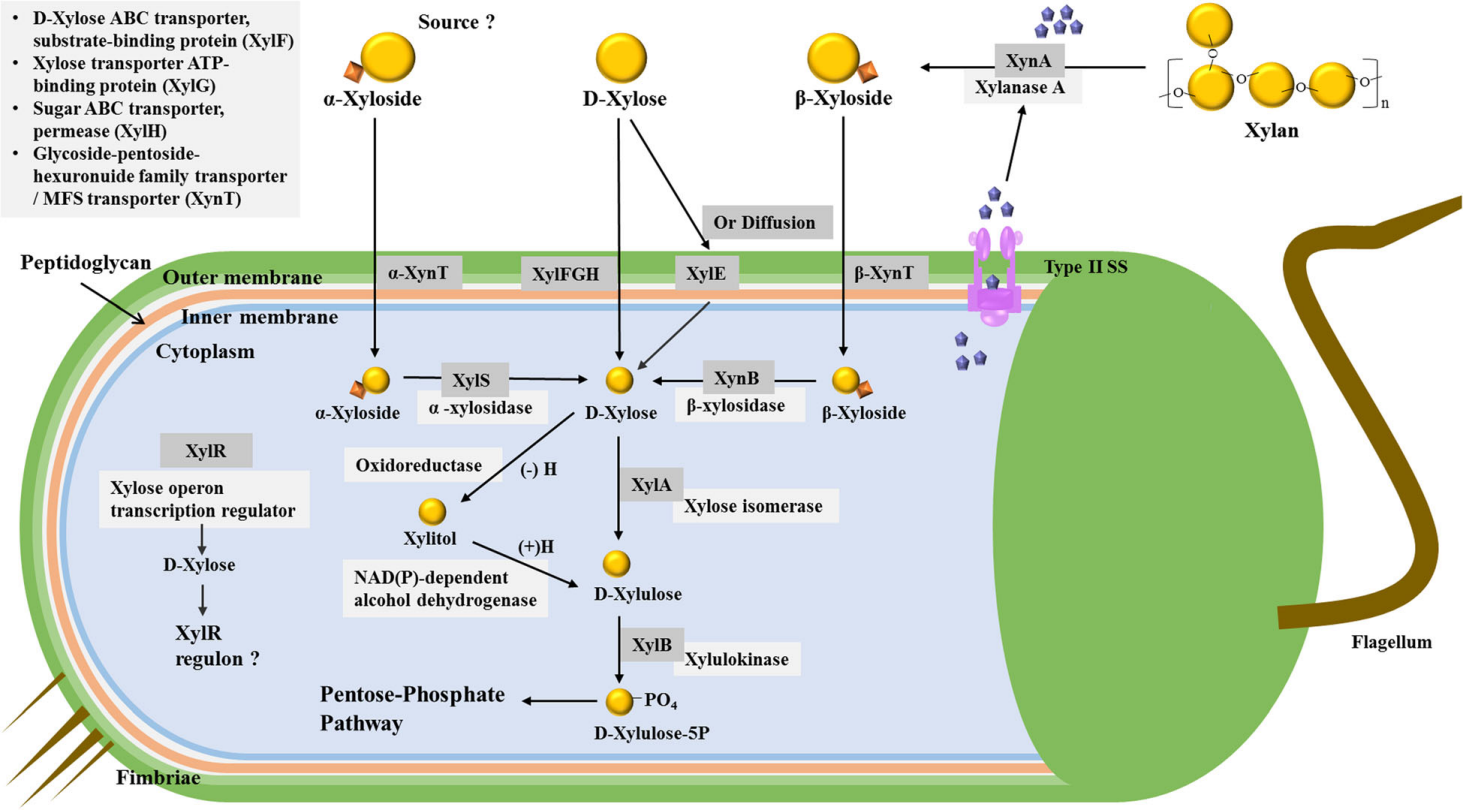
Schematic representation of xylose utilization by *C. sakazakii* strain MOD1_Jor22. The proposed model for xylose utilization involves activation of the xylose regulon by the binding of D-xylose with XylR. It is thought that D-xylose enters the cell either through diffusion or transport via XylE or XylFGH. In addition, xylanase A (XynA) is secreted to the extracellular milieu through an unknown type 2 secretion component where it can digest xylan to β-xyloside which is then brought into the cell via a xyloside transporter (XynT, a putative β-xyloside transporter) where XynB (β-xylosidase) converts it to D-xylose. Though unconfirmed, α- xyloside is thought to be transported into the cell where XylS (α-xylosidase) converts to D- xylose. D-xylose then is converted to D-xylulose by XylA (xylose isomerase) and then converted to D-xylulose-5P by XylB (xylulokinase). This physiological pathway is identical to that of *E. coli*. Similar to that of *E. coli*, *Cronobacter* also have anaerobic metabolic pathway where D- xylose is converted to xylitol by oxidoreductase and then converted to D-xyloulose using NAD(P)-dependent alcohol dehydrogenase. D-xylulose-5P is then shunted into pentose- phosphate pathway

Figure 4 illustrates the proposed molecular basis of how *C. sakazakii* (strain MOD1_Jor22 as an example) may utilize D-xylose, xylose-containing plant cell wall polymers (xylans, hemicellulose-like, and cellulose) or α- and β-xylosides. D-xylose enters the cytoplasm of a cell either by diffusion or by transport and binds to the AraC-like positive xylose operon transcription regulator, *XylR*. *XylR* is, identical to AraC which activates the transcription of the analogous arabinose utilization operon, *araBAD*, *araE* and *araFGH* operons, but represses the transcription of the *araC* operon. Once bound, *XylR* actuates the xylose regulon by activating the transcription of the *xylFGH*, *xylR*, *xylAB*, and *xylE* genes. In fact, in *E. coli*, the xylose transporters *XylE* and *XylFGH* can transport both arabinose and xylose; conversely the arabinose transporters *Ara**E* and *Ara**FGH* can take up xylose, even in the absence of arabinose [56]. As with arabinose, expression of the *XylE* and *XylFGH* transporters increases the rate of xylose uptake and further enhances activation of the regulon. Another set of genes, which are also outside the operon, may be triggered through the proposed activation of the xylose regulon: *xynA* encoding for Xylanase A (*xynA*, locus tag C5934_19110) which is an Endo-1,4-β-xylanase and may be secreted by a proposed type 2 secretion system. A third pathway of xylose utilization, also seen in *E. coli,* was found in these *Cronobacter* spice strain’s genomes and includes a xylulose reductase, an oxidoreductase (locus tag C5934_08370), and a NAD(P)-dependent alcohol dehydrogenase (locus tag C5934_08415) which are thought to be activated under anaerobic growth conditions [56]. D-xylose, or transported α/β-xylosides (via α/β-XynTs) are converted to D-xylose by α/β-xylosidases (XylS/XynB) within the cell. It is not certain, at this time, how xylans are converted to α-xylosides in the extracellular milieu. However, the fact *Cronobacter* possess an α-xylosidases (*xylS*) and an adjacent *xynT* gene, suggests that that α- xylosides may be transported into the cell and then converted to D-Xylose, which is then converted to D-xylulose by xylose isomerase (*XylA*) and then phosphorylated by Xylulose kinase (*XylB*). Then, xylulose 5-phosphate is metabolized by the enzymes of the pentose phosphate pathway [56]. Together these results support those reported by Srikumar et al. [30], which suggest that 5-carbon sugar physiological mechanisms utilized by *Cronobacter* plays important roles in its overall survival strategy.

## Conclusions

Several lines of evidence posited by Schmid et al. [17] and Joseph et al. [32] suggest that the ancestral econiche for *Cronobacter* species may have been eukaryotic plants. It is interesting to speculate that both the survival mechanisms, which we now recognize through the use of NGS and the study of efflux of important molecules such as sugars, osmoprotectants and metal ions gives us insights into the processes that we hypothesize may also allow *Cronobacter* to survive desiccation, as well as, cause human illness [29]. Although these processes may very well be genomic remnants from when the hypothetical ancestral *Cronobacter* species was evolving approximately 59 million years ago (during the Palaeogene geologic period), information proffered in this report by no means represents the total genomic story of *C. sakazakii*. We hope that it offers glimpses or insights into the genomic complexity of this important foodborne pathogen.

## Additional files

Additional file 1: **Table S1.** Individual genome statistics of the *C. sakazakii* strains which were evaluated in the study. Data include genome size, CDS, number of scafolds, CDSs, Protein coding, RNA and Pseudo genes, Genes in internal clusters, genes assigned to COGs, Genes with predicted Pfam, signal peptides, and transmembrane protein domains, and CRISPR repeats^a,b^. (PDF 385 kb)

Additional file 2: **Table S2.** Number of proteins per COG category present in each individual spice-origin *C. sakazakii* strain^a^. (XLSX 16 kb)

Additional file 3: **Table S3.** Summary table of spice-origin *C. sakazakii* protein locus tag IDs identified by NCBI’s PGAP annotation pipeline. (XLSX 4454 kb)

Additional file 4: **Table S4.** Summary table of pESA3-like RAST gene IDs, contig, % identity, and annotations associated with spice-origin *C. sakazakii* genomes. (XLSX 166 kb)

Additional file 5: **Table S5.** Summary table of sequence repeats (inverted repeat, direct repeat, and palindrome) that are associated with the xylose untilization operon of spice-origin *C. sakazakii* genomes evaluated in the study*. (XLSX 14 kb)

## Abbreviations

MIGS: Minimum information about a genome sequence
NAS: Non-traceable author statement
TAS: Traceable author statement

## References

[1] Tall BD, Chen Y, Yan QQ, Gopinath GR, Grim CJ, Jarvis KG, Fanning S, Lampel KA (2014). *Cronobacter*: an emergent pathogen a using meningitis to neonates through their feeds. Sci Prog.

[2] Iversen C, Mullane N, McCardell B, Tall BD, Lehner A, Fanning S, Stephan R, Joosten H (2008). *Cronobacter* gen. Nov., a new genus to accommodate the biogroups of *Enterobacter sakazakii*, and proposal of *Cronobacter sakazakii* gen. Nov., comb. nov., *Cronobacter malonaticus* sp. nov., *Cronobacter turicensis* sp. nov., *Cronobacter muytjensii* sp. nov., *Cronobacter dublinensis* sp. nov., *Cronobacter* genomospecies 1 and of three subspecies, *Cronobacter dublinensis* subsp. *dublinensis* subsp. nov., *Cronobacter dublinensis* subsp. *lausannensis* subsp. nov. and *Cronobacter dublinensis* subsp. *lactaridi* subsp. nov. Int J Syst Evol Microbiol.

[3] Joseph S, Cetinkaya E, Drahovska H, Levican A, Figueras MJ, Forsythe SJ (2012). *Cronobacter condimenti* sp. nov., isolated from spiced meat and *Cronobacter universalis* sp. nov., a species designation for *Cronobacter* sp. genomospecies 1, recovered from a leg infection, water and food ingredients. Int J Syst Evol Microbiol.

[4] Hunter CJ, Petrosyan M, Ford HR, Prasadarao NV (2008). *Enterobacter sakazakii*: an emerging pathogen in infants and neonates. Surg Infect.

[5] Holý O, Petrželová J, Hanulík V, Chromá M, Matoušková I, Forsythe SJ (2014). Epidemiology of *Cronobacter* spp. isolates from patients admitted to the Olomouc University hospital (Czech Republic). Epidemiol Mikrobiol Imunol.

[6] Alsonosi A, Hariri S, Kajsík M, Oriešková M, Hanulík V, Röderová M, Petrželová J, Kollárová H, Drahovská H, Forsythe S, Holý O (2015). The speciation and genotyping of *Cronobacter* isolates from hospitalised patients. Eur J Clin Microbiol Infect Dis.

[7] Patrick ME, Mahon BE, Greene SA, Rounds J, Cronquist A, Wymore K, Boothe E, Lathrop S, Palmer A, Bowen A. Incidence of *Cronobacter* spp. infections, United States, 2003–2009. Emerg Infect Dis. 2014;20:1520–3. 10.3201/eid2009.140545 PMC4178417 25148394

[8] Himelright I, Harris E, Lorch V, Anderson M, Jones T, Craig A, Kuehnert M, Forster T, Arduino M, Jensen B, Jernigan D (2002). *Enterobacter sakazakii* infections associated with the use of powdered infant formula–Tennessee, 2001. Morb Mortal Wkly Rep.

[9] Jason J (2012). Prevention of invasive *Cronobacter* infections in young infants fed powdered infant formulas. Pediatrics.

[10] FAO/WHO. *Enterobacter sakazakii* and other micro-organisms in powdered infant formula: meeting report, Microbiological Risk Assessment Series. Rome; 2004. p. 6. http://www.fao.org/3/a-y5502e.pdf.

[11] Noriega FR, Kotloff KL, Martin MA, Schwalbe RS (1990). Nosocomial bacteremia caused by *Enterobacter sakazakii* and *Leuconostoc mesenteroides* resulting from extrinsic contamination of infant formula. Pediatric Infect Dis J.

[12] Berthold-Pluta A, Garbowska M, Stefańska I, Pluta A. Microbiological quality of selected ready-to-eat leaf vegetables, sprouts and non-pasteurized fresh fruit-vegetable juices including the presence of Cronobacter spp. Food Microbiol. 2017;65:221–30. 10.1016/j.fm.2017.03.005. 10.1016/j.fm.2017.03.005 28400006

[13] Sani NA, Odeyemi OA (2015). Occurrence and prevalence of *Cronobacter* spp. in plant and animal derived food sources: a systematic review and meta-analysis. Springerplus.

[14] Lou X, Si G, Yu H, Qi J, Liu T, Fang Z (2014). Possible reservoir and routes of transmission of *Cronobacter* (*Enterobacter sakazakii*) via wheat flour. Food Control.

[15] Kandhai MC, Heuvelink AE, Reij MW, Beumer RR, Dijk R, van Tilburg JJHC, van Schothorst M, Gorris LGM (2010). A study into the occurrence of *Cronobacter* spp. in the Netherlands between 2001 and 2005. Food Control.

[16] Chase HR, Eberl L, Stephan R, Jeong H, Lee C, Finkelstein S, Negrete F, Gangiredla J, Patel I, Tall BD, Gopinath GR, Lehner A (2017). Draft genome sequence of *Cronobacter sakazakii* GP1999, sequence type 145, an epiphytic isolate obtained from the tomato’s rhizoplane/rhizosphere continuum. Genome Announc.

[17] Schmid M, Iversen C, Gontia I, Stephan R, Hofmann A, Hartmann A, Jha B, Eberl L, Riedel K, Lehner A (2009). Evidence for a plant-associated natural habitat for *Cronobacter* spp. Res Microbiol.

[18] Gopinath GR, Chase HR, Gangiredla J, Eschwar A, Jang H, Patel I, Negrete F, Finkelstein S, Park E, Chung T, Yoo Y, Woo J, Lee Y, Park J, Choi H, Jeong S, Jun S, Kim M, Lee C, Jeong H, Fanning S, Stephan R, Iversen C, Reich F, Klein G, Lehner A, Tall BD (2017). Genomic characterization of malonate positive *Cronobacter sakazakii* serotype O:2, sequence type 64 strains, isolated from clinical, food, and environment samples. Gut Pathogens.

[19] Jang H, Addy N, Ewing L, Beaubrun JJG, Lee Y, Woo J, Negrete F, Finkelstein S, Tall BD, Lehner A, Eshwar A, Gopinath GR (2018). Whole genome sequences of *Cronobacter sakazakii* isolates obtained from plant-origin foods and dried food manufacturing environments. Genome Announc.

[20] Jaradat ZW, Ababneh QO, Saadoun IM, Samara NA, Rashdan AM (2009). Isolation of *Cronobacter* spp. (formerly *Enterobacter sakazakii*) from infant food, herbs and environmental samples and the subsequent identification and confirmation of the isolates using biochemical, chromogenic assays, PCR and 16S rRNA sequencing. BMC Microbiol.

[21] Chon JW, Song KY, Kim SY, Hyeon JY, Seo KH (2012). Isolation and characterization of *Cronobacter* from desiccated foods in Korea. J Food Sci.

[22] Urmenyi AMC, Franklin AW (1961). Neonatal death from pigmented coliform infection. Lancet.

[23] Farmer IIIJJ, Asbury MA, Hickman FW, Brenner DJ, the Enterobacteriaceae Study Group (1980). *Enterobacter sakazakii*: a new species of “*Enterobacteriaceae*” isolated from clinical specimens. Int J Syst Bacteriol.

[24] Breeuwer P, Lardeau A, Peterz M, Joosten HM (2003). Desiccation and heat tolerance of *Enterobacter sakazakii*. J Appl Microbiol.

[25] Shaker RR, Osaili TM, Abu Al-Hasan AS, Ayyash MM, Forsythe SJ (2008). Effect of desiccation, starvation, heat, and cold stresses on the thermal resistance of *Enterobacter sakazakii* in rehydrated infant milk formula. J Food Sci.

[26] Osaili T, Forsythe S (2009). Desiccation resistance and persistence of *Cronobacter* species in infant formula. Int J Food Microbiol.

[27] Walsh D, Molloy C, Iversen C, Carroll J, Cagney C, Fanning S, Duffy G (2011). Survival characteristics of environmental and clinically derived strains of *Cronobacter sakazakii* in infant milk formula (IMF) and ingredients. J Appl Microbiol.

[28] Yan Q, Power KA, Cooney S, Fox E, Gopinath GR, Grim CJ, Tall BD, McCusker MP, Fanning S (2013). Complete genome sequence and phenotype microarray analysis of *Cronobacter sakazakii* SP291: a persistent isolate cultured from a powdered infant formula production facility. Front Microbiol.

[29] Lehner A, Tall BD, Fanning S, Shabarinath S. *Cronobacter* spp. – opportunistic foodborne pathogens: an update on evolution, osmotic adaptation and pathogenesis. Curr Clin Micro Rpt. 2018. 10.1007/s40588-018-0089-7.

[30] Srikumar S, Cao Y, Yan Q, Van Hoorde K, Nguyen S, Cooney S, Gopinathrao GR, Tall BD, Sivasankaran SK, Lehner A, Stephan R, Fanning S. RNA sSequencing based transcriptional overview of xerotolerance in Cronobacter sakazakii. Appl.Environ Microbiol. 2018.10.1128/AEM.01993-18. [Epub ahead of print]. 10.1128/AEM.01993-18 PMC6344630 30446557

[31] Forsythe SJ (2018). Updates on the *Cronobacter* genus. Annu Rev Food Sci Technol.

[32] Joseph S, Sonbol H, Hariri S, Desai P, McClelland M, Forsythe SJ (2012). Diversity of the *Cronobacter* genus as revealed by multilocus sequence typing. J Clin Microbiol.

[33] Jolley KA, Maiden MCJ (2010). BIGSdb: scalable analysis of bacterial genome variation at the population level. BMC Bioinformatics.

[34] Field D, Garrity G, Gray T, Morrison N, Selengut J, Sterk P (2008). The minimum information about a genome sequence (MIGS) specification. Nat Biotechnol.

[35] Grim CJ, Kotewicz ML, Power KA, Gopinath G, Franco AA, Jarvis KG, Yan QQ, Jackson SA, Sathyamoorthy V, Hu L, Pagotto F, Iversen C, Lehner A, Stephan R, Fanning S, Tall BD (2013). Pan-genome analysis of the emerging foodborne pathogen *Cronobacter* spp. suggests a species-level bidirectional divergence driven by niche adaptation. BMC Genomics.

[36] Chase HR, Gopinath GR, Eshwar AK, Stoller A, Fricker-Feer C, Gangiredla J, Patel IR, Cinar HN, Jeong H, Lee C, Negrete F, Finkelstein S, Stephan R, Tall BD, Lehner A (2017). Comparative genomic characterization of the highly persistent and potentially virulent *Cronobacter sakazakii* ST83, CC65 strain H322 and other ST83 strains. Front Microbiol.

[37] Aziz RK, Bartels D, Best AA, DeJongh M, Disz T, Edwards RA, Formsma K, Gerdes S, Glass EM, Kubal M, Meyer F, Olsen GJ, Olson R, Osterman AL, Overbeek RA, McNeil LK, Paarmann D, Paczian T, Parrello B, Pusch GD, Reich C, Stevens R, Vassieva O, Vonstein V, Wilke A, Zagnitko O (2008). The RAST server: rapid annotations using subsystems technology. BMC Genomics.

[38] Allard MW, Strain E, Melka D, Bunning K, Musser SM, Brown EW, Timme R (2016). Practical value of food pathogen traceability through building a whole genome sequencing network and database. J Clin Microbiol.

[39] Allard MW, Bell R, Ferreira CM, Gonzalez-Escalona N, Hoffmann M, Muruvanda T, Ottesen A, Ramachandran P, Reed E, Sharma S, Stevens E, Timme R, Zheng J, Brown EW (2018). Genomics of foodborne pathogens for microbial food safety. Curr Opin Biotechnol.

[40] Huerta-Cepas J, Szklarczyk D, Forslund K, Cook H, Heller D, Walter MC, Rattei T, Mende DR, Sunagawa S, Kuhn M, Jensen LJ, von Mering C, Bork P (2016). eggNOG 4.5: a hierarchical orthology framework with improved functional annotations for eukaryotic, prokaryotic and viral sequences. Nucleic Acids Res.

[41] Tatusov RL, Koonin EV, Lipman DJ (1997). A genomic perspective on protein families. Science.

[42] Ashburner M, Ball CA, Blake JA, Botstein D, Butler H, Cherry JM (2000). Gene ontology: tool for the unification of biology. Nat Genet.

[43] Franco AA, Hu L, Grim CJ, Gopinath G, Sathyamoorthy V, Jarvis KG, Lee C, Sadowski J, Kim J, Kothary MH, McCardell BA, Tall BD (2011). Characterization of putative virulence genes on the related RepFIB plasmids harbored by *Cronobacter* spp. Appl Environ Microbiol.

[44] Kucerova E, Clifton SW, Xia XQ, Long F, Porwollik S, Fulton L, Fronick C, Minx P, Kyung K, Warren W, Fulton R, Feng D, Wollam A, Shah N, Bhonagiri V, Nash WE, Hallsworth-Pepin K, Wilson RK, McClelland M, Forsythe SJ (2010). Genome sequence of *Cronobacter sakazakii* BAA-894 and comparative genomic hybridization analysis with other *Cronobacter* species. PLoS One.

[45] Power KA, Yan Q, Fox EM, Cooney S, Fanning S (2013). Genome sequence of *Cronobacter sakazakii* SP291, a persistent thermotolerant isolate derived from a factory producing powdered infant formula. Genome Announc.

[46] García-Calderón CB, Casadesús J, Ramos-Morales F (2007). Rcs and PhoPQ regulatory overlap in the control of *Salmonella* enterica virulence. J Bacteriol.

[47] Stephan R, Lehner A, Tischler P, Rattei T (2011). Complete genome sequence of *Cronobacter turicensis* LMG 23827, a food-borne pathogen causing deaths in neonates. J Bacteriol.

[48] Gardner SN, Slezak T, Hall BG (2015). kSNP3.0: SNP detection and phylogenetic analysis of genomes without genome alignment or reference genome. Bioinformatics.

[49] Bontemps-Gallo S, Lacroix JM (2015). New insights into the biological role of the osmoregulated periplasmic glucans in pathogenic and symbiotic bacteria. Environ Microbiol Rep.

[50] Walker G, Hertle R, Braun V (2004). Activation of *Serratia marcescens* hemolysin through a conformational change. Infect Immun.

[51] Cruz A, Xicohtencatl-Cortes J, González-Pedrajo B, Bobadilla M, Eslava C, Rosas I (2011). Virulence traits in *Cronobacter* species isolated from different sources. Can J Microbiol.

[52] Gunn JS, Alpuche-Aranda CM, Loomis WP, Belden WJ, Miller SI (1995). Characterization of the *Salmonella typhimurium pagC*/*pagD* chromosomal region. J Bacteriol.

[53] Olofsson K, Bertilsson M, Lidén G (2008). A short review on SSF – an interesting process option for ethanol production from lignocellulosic feedstocks. Biotechnol Biofuels.

[54] Santos CR, Hoffmam ZB, de Matos Martins VP, Zanphorlin LM, de Paula Assis LH, Honorato RV, Lopes de Oliveira PS, Ruller R, Murakami MT (2014). Molecular mechanisms associated with xylan degradation by *Xanthomonas* plant pathogens. J Biol Chem.

[55] Mansfield J, Genin S, Magori S, Citovsky V, Sriariyanum M, Ronald P, Dow M, Verdier V, Beer SV, Machado MA, Toth I, Salmond G, Foster GD (2012). Top 10 plant pathogenic bacteria in molecular plant pathology. Mol Plant Pathol.

[56] Luo Y, Zhang T, Wu H (2014). The transport and mediation mechanisms of the common sugars in *Escherichia coli*. Biotechnol Adv.

[57] Carter MQ, Xue K, Brandl MT, Liu F, Wu L, Louie JW, Mandrell RE, Zhou J (2012). Functional metagenomics of *Escherichia coli* O157:H7 interactions with spinach indigenous microorganisms during biofilm formation. PLoS One.

[58] Crozier L, Hedley PE, Morris J, Wagstaff C, Andrews SC, Toth I, Jackson RW, Holden NJ (2016). Whole-transcriptome analysis of verocytotoxigenic *Escherichia coli* O157:H7 (Sakai) suggests plant-species-specific metabolic responses on exposure to spinach and lettuce extracts. Front Microbiol.

[59] de Castro SS, de Castro OL, Jaiswal AK, Azevedo V (2016). Genomic Islands: an overview of current software tools and future improvements. J Integ Bioinform.

[60] Juhas M, van der Meer JR, Gaillard M, Harding RM, Hood DW, Crook DW (2009). Genomic islands: tools of bacterial horizontal gene transfer and evolution. FEMS Microbiol Rev.

[61] Baek D, Nam J, Koo YD, Kim DH, Lee J, Jeong JC, Kwak SS, Chung WS, Lim CO, Bahk JD, Hong JC, Lee SY, Kawai-Yamada M, Uchimiya H, Yun DJ (2004). Bax-induced cell death of Arabidopsis is meditated through reactive oxygen-dependent and -independent processes. Plant Mol Biol.

[62] Chen Y, Lampel K, Hammack T, U.S. FDA (2012). Bacteriological analytical manual. Chapter 29. Cronbacter.

[63] Chen Y, Noe KE, Thompson S, Elems CA, Brown EW, Lampel KA, Hammack TS (2012). Evaluation of a revised U.S. Food and Drug Administration method for the detection of *Cronobacter* in powdered infant formula: a collaborative study. J Food Prot.

[64] International Organization for Standardization (ISO) (2017). Microbiology of the food chain – horizontal method for the detection of *Cronobacter* spp. ISO/TS 22964.

[65] Saitou N, Nei M (1987). The neighbor-joining method: a new method for reconstructing phylogenetic trees. Mol Biol Evol.

[66] Tamura K, Nei M, Kumar S (2004). Prospects for inferring very large phylogenies by using the neighbor-joining method. Proc Natl Acad Sci U S A.

[67] Kumar S, Stecher G, Tamura K (2016). MEGA7: molecular evolutionary genetics analysis version 7.0 for bigger datasets. Mol Biol Evol.

[68] Felsenstein J (1985). Confidence limits on phylogenies: an approach using bootstrap. Evolution.

