# Supplementary material for: Draft genomes of Cronobacter sakazakii strains isolated from dried spices bring unique insights into the diversity of plant-associated strains
Source: Stand Genomic Sci. 2018 Nov 29;13:35. doi: 10.1186/s40793-018-0339-6 (PMC6267090; doi:10.1186/s40793-018-0339-6)
Supplement: Supplementary file 1 — Table S1. Individual genome statistics of the C. sakazakii strains which were evaluated in the study. Data include genome size, CDS, number of scafolds, CDSs, Protein coding, RNA and Pseudo genes, Genes in internal clusters, genes assigned to COGs, Genes with predicted Pfam, signal peptides, and transmembrane protein domains, and CRISPR repeatsa,b. (PDF 385 kb) [file 40793_2018_339_MOESM1_ESM.pdf]

Table S1. Individual genome statistics of the *C.sakazakii* strains which were evaluated in the study. Data include genome size, CDS, number of scafolds, CDSs, Protein coding, RNA and Pseudo genes, Genes in internal clusters, genes assigned to COGs, Genes with predicted Pfam, signal peptides, and transmembrane protein domains, and CRISPR repeats.[a,b]

	Strains													
	MOD1_O23mB		MOD1_788569		MOD1_AS-2		MOD1_AS-4		MOD1_AS-13		MOD1_AS-15		MOD1_O21_16	
**Attributes**	value	% of total	value	% of total	value	% of total	value	% of total	value	% of total	value	% of total	value	% of total
Genome size (kb)	4,337.0	100.0	4,052.0	100.0	4,303.0	100.0	4,279.0	100.0	4,305.0	100.0	4,311.0	100.0	4,405.0	100.0
DNA coding (kb)	3,827.0	88.3	3,980.0	88.4	3,801.0	88.3	3,779.0	88.3	3,802.0	88.3	3,807.0	88.3	3,907.0	88.7
DNA G + C (kb)	2,469.0	56.9	2,556.0	56.8	2,453.0	57.0	2,438.0	57.0	2,454.0	57.0	2,457.0	57.0	2,512.0	57.0
DNA scaffolds	44.0	100.0	29.0	100.0	45.0	100.0	53.0	100.0	35.0	100.0	40.0	100.0	31.0	100.0
Total genes	4,135.0	100.0	4,282.0	100.0	4,093.0	100.0	4,094.0	100.0	4,094.0	100.0	4,106.0	100.0	4,168.0	100.0
Protein coding genes	3,985.0	96.4	4,125.0	96.3	3,944.0	96.4	3,940.0	96.2	3,946.0	96.4	3,955.0	96.3	4,015.0	96.3
RNA genes	150.0	3.6	157.0	3.7	149.0	3.6	154.0	3.8	148.0	3.6	151.0	3.7	153.0	3.7
Pseudo genes*	115.0	-**	119.0	-	82.0	-	83.0	-	80.0	-	82.0	-	75.0	-
Genes in internal clusters	850.0	20.6	962.0	22.5	889.0	21.7	880.0	21.5	892.0	21.8	892.0	21.7	885.0	21.2
Genes assigned to COGs	3,102.0	75.0	3,194.0	74.6	3,123.0	76.3	3,105.0	75.8	3,128.0	76.4	3,133.0	76.3	3,167.0	76.0
Genes with Pfam domain	3,614.0	87.4	3,747.0	87.5	3,618.0	88.4	3,605.0	88.1	3,619.0	88.4	3,627.0	88.3	3,680.0	88.3
Genes with signal peptides	407.0	9.8	425.0	9.9	411.0	10.0	407.0	9.9	412.0	10.1	415.0	10.1	403.0	9.7
Genes with transmembrane protiens	983.0	23.8	1,003.0	23.4	983.0	24.0	979.0	23.9	983.0	24.0	987.0	24.0	993.0	23.8
CRISPR repeats*	3.0	-**	3.0	-	2.0	-	2.0	-	2.0	-	2.0	-	2.0	-

[a] Data was obtained from the JGI IMG pipeline.

[b] A summary of the genome statistics for these strain is given Table 4.

* Data was obtained from the NCBI, https://www.ncbi.nlm.nih.gov/nuccore.

** NCBI did not have the percentage of total number of pseudo genes and CRISPR repeats.

Table S1 continued

	Strains													
	MOD1_Jor20		MOD1_Jor22		MOD1_Jor44		MOD1_Jor93		MOD1_Jor96		MOD1_Jor103		MOD1_Jor146	
**Attributes**	value	% of total	value	% of total	value	% of total	value	% of total	value	% of total	value	% of total	value	% of total
Genome size (kb)	4,466.0	100.0	4,467.0	100.0	4,478.0	100.0	4,326.0	100.0	4,666.0	100.0	4,315.0	100.0	4,404.0	100.0
DNA coding (kb)	3,931.0	88.0	3,934.0	88.1	3,963.0	88.5	3,823.0	88.4	4,118.0	88.3	3,812.0	88.4	3,895.0	88.4
DNA G + C (kb)	2,534.0	56.8	2,534.0	56.7	2,549.0	56.9	2,468.0	57.1	2,639.0	56.6	2,461.0	57.0	2,508.0	56.9
DNA scaffolds	68.0	100.0	46.0	100.0	36.0	100.0	39.0	100.0	36.0	100.0	83.0	100.0	36.0	100.0
Total genes	4,285.0	100.0	4,273.0	100.0	4,277.0	100.0	4,100.0	100.0	4,471.0	100.0	4,106.0	100.0	4,168.0	100.0
Protein coding genes	4,136.0	96.5	4,126.0	96.6	4,129.0	96.5	3,942.0	96.2	4,316.0	96.5	3,955.0	96.3	4,019.0	96.4
RNA genes	149.0	3.5	147.0	3.4	148.0	3.5	158.0	3.9	155.0	3.5	151.0	3.7	149.0	3.6
Pseudo genes*	123.0	-	123.0	-	86.0	-	100.0	-	112.0	-	157.0	-	73.0	-
Genes in internal clusters	882.0	20.6	886.0	20.7	885.0	20.7	841.0	20.5	947.0	21.2	829.0	20.2	901.0	21.6
Genes assigned to COGs	3,137.0	73.2	3,141.0	73.5	3,173.0	74.2	3,115.0	76.0	3,241.0	72.5	3,101.0	75.5	3,165.0	75.9
Genes with Pfam domain	3,702.0	86.4	3,697.0	86.5	3,713.0	86.8	3,595.0	87.7	3,849.0	86.1	3,597.0	87.6	3,678.0	88.2
Genes with signal peptides	415.0	9.7	416.0	9.7	409.0	9.6	408.0	10.0	427.0	9.6	404.0	9.8	413.0	9.9
Genes with transmembrane protiens	1,010.0	23.6	1,009.0	23.6	996.0	23.3	981.0	23.9	1,015.0	22.7	978.0	23.8	992.0	23.8
CRISPR repeats*	4.0	-	3.0	-	3.0	-	3.0	-	2.0	-	2.0	-	4.0	-

Table S1 continued

	Strains													
	MOD1_Jor148		MOD1_Jor151		MOD1_Jor154		MOD1_Jor172		MOD1_Jor173		MOD1_Jor178		MOD1_Jor183	
**Attributes**	value	% of total	value	% of total	value	% of total	value	% of total	value	% of total	value	% of total	value	% of total
Genome size (kb)	4,568.0	100.0	4,481.0	100.0	4,387.0	100.0	4,330.0	100.0	4,375.0	100.0	4,716.0	100.0	4,320.0	100.0
DNA coding (kb)	4,023.0	88.1	3,966.0	88.5	3,873.0	88.3	3,827.0	88.4	3,869.0	88.4	4,160.0	88.2	3,818.0	88.4
DNA G + C (kb)	2,593.0	56.8	2,550.0	56.9	2,499.0	57.0	2,468.0	57.0	2,490.0	56.9	2,664.0	56.5	2,460.0	56.9
DNA scaffolds	92.0	100.0	34.0	100.0	74.0	100.0	23.0	100.0	32.0	100.0	65.0	100.0	100.0	100.0
Total genes	4,425.0	100.0	4,282.0	100.0	4,191.0	100.0	4,131.0	100.0	4,110.0	100.0	4,541.0	100.0	4,090.0	100.0
Protein coding genes	4,275.0	96.6	4,135.0	96.6	4,049.0	96.9	3,969.0	96.1	3,957.0	96.3	4,383.0	96.5	3,937.0	96.3
RNA genes	150.0	3.4	147.0	3.4	142.0	3.4	162.0	3.9	153.0	3.7	158.0	3.5	153.0	3.7
Pseudo genes*	118.0	-	89.0	-	111.0	-	83.0	-	84.0	-	130.0	-	97.0	-
Genes in internal clusters	901.0	20.4	882.0	20.6	883.0	21.1	891.0	21.6	879.0	21.4	955.0	21.0	862.0	21.1
Genes assigned to COGs	3,203.0	72.4	3,178.0	74.2	3,146.0	75.1	3,140.0	76.0	3,157.0	76.8	3,251.0	71.6	3,102.0	75.8
Genes with Pfam domain	3,806.0	86.0	3,714.0	86.7	3,679.0	87.8	3,648.0	88.3	3,642.0	88.6	3,879.0	85.4	3,603.0	88.1
Genes with signal peptides	409.0	9.2	410.0	9.6	411.0	9.8	411.0	10.0	420.0	10.2	431.0	9.5	417.0	10.2
Genes with transmembrane protiens	1,011.0	22.9	996.0	23.3	987.0	23.6	986.0	23.9	981.0	23.9	1,023.0	22.5	983.0	24.0
CRISPR repeats*	2.0	-	3.0	-	2.0	-	2.0	-	4.0	-	2.0	-	4.0	-

Table S1 continued

	**Strains**									
	MOD1_KW3		MOD1_KW13		MOD1_O21_13		MOD1_O26_1		MOD1_O26_4	
**Attributes**	value	% of total	value	% of total	value	% of total	value	% of total	value	% of total
Genome size (kb)	4,369.0	100.0	4,449.0	100.0	4,374.0	100.0	4,408.0	100.0	4,338.0	100.0
DNA coding (kb)	3,857.0	88.3	3,962.0	88.3	3,877.0	88.7	3,910.0	88.7	3,828.0	88.3
DNA G + C (kb)	2,487.0	56.9	2,552.0	56.9	2,493.0	57.0	2,514.0	57.0	2,469.0	56.9
DNA scaffolds	40.0	100.0	30.0	100.0	32.0	100.0	35.0	100.0	23.0	100.0
Total genes	4,154.0	100.0	4,329.0	100.0	4,163.0	100.0	4,171.0	100.0	4,131.0	100.0
Protein coding genes	4,002.0	96.3	4,174.0	96.4	4,021.0	96.6	4,019.0	96.4	3,973.0	96.2
RNA genes	152.0	3.7	155.0	3.6	142.0	3.4	152.0	3.6	158.0	3.8
Pseudo genes*	119.0	-	110.0	-	78.0	-	76.0	-	111.0	-
Genes in internal clusters	889.0	21.4	875.0	20.2	889.0	21.4	886.0	21.2	853.0	20.7
Genes assigned to COGs	3,150.0	75.8	3,141.0	72.6	3,146.0	75.6	3,171.0	76.0	3,108.0	75.2
Genes with Pfam domain	3,640.0	87.6	3,700.0	85.5	3,677.0	88.3	3,678.0	88.2	3,605.0	87.3
Genes with signal peptides	415.0	10.0	436.0	10.1	410.0	9.9	403.0	9.7	407.0	9.9
Genes with transmembrane protiens	997.0	24.0	1,038.0	24.0	991.0	23.8	991.0	23.8	984.0	23.8
CRISPR repeats*	2.0	-	2.0	-	2.0	-	2.0	-	3.0	-
